# A Constitutional Mismatch Repair Deficiency Syndrome Presented With an Advanced Rectal Cancer in a Juvenile Female: A Case Report and Literature Review

**DOI:** 10.7759/cureus.24615

**Published:** 2022-04-30

**Authors:** Mohammed N AlAli, Abdulrahman H Zikry, Sulaiman A AlShammari, Mohammed Ayesh Zayed, Mohammed Alswayyed, Omar A AlObeed

**Affiliations:** 1 Department of Surgery, Prince Mohammed bin Abdulaziz Hospital, Second Health Cluster in Central Region, Ministry of Health, Riyadh, SAU; 2 Department of Surgery, College of Medicine, King Khalid University Hospital, King Saud University, Riyadh, SAU; 3 Department of Radiology, King Khalid University Hospital, King Saud University, Riyadh, SAU; 4 Department of Pathology and Laboratory Medicine, College of Medicine, King Khalid University Hospital, King Saud University, Riyadh, SAU

**Keywords:** genetic disorders, lynch related, rectal mass, rectal cancer, cmmrd syndrome

## Abstract

The constitutional mismatch repair deficiency (CMMRD) syndrome is a rare and challenging condition with a poor prognosis. It results from biallelic mismatch repair gene mutations and leads to multiorgan cancers. Therefore, we report the first case of advanced juvenile rectal cancer related to CMMRD syndrome in the Gulf region. She is a 13-year-old female, born to non-consanguineous parents with a positive family history of malignancy, presented with an eight-month history of a retractable bulging anal mass associated with diarrhea mixed with blood and constitutional symptoms. She was cachectic with café au lait spots all over her body. Upon investigation, she was found to have invasive rectal adenocarcinoma. The case was started on neoadjuvant chemoradiotherapy in addition to genetic testing which showed a homozygous pathogenic variant in PMS2, indicating CMMRD syndrome. The patient underwent pre-operative post-neoadjuvant reassessment followed by laparoscopic total proctocolectomy with ileal J-pouch creation and ileoanal anastomosis with temporary diverting loop ileostomy which later on was reversed with no complications or recurrence. The family declined to continue the adjuvant therapy but accepted the surveillance programs and genetic testing. Unusual or late presentation secondary to a very rare syndrome like CMMRD is a major challenge to clinicians, hence a high index of suspicion and proper utilization of genetics programs might be the best available solutions.

## Introduction

Colorectal cancer (CRC) during childhood is one of the red flags to consider genetic disorders in the affected patient as well as his family members. Lynch syndrome (LS) is a well-known risk factor for hereditary CRC with an autosomal dominant mutation in one of the DNA genes in young adults if compared to the constitutional mismatch repair deficiency (CMMRD) syndrome which is known as a rare autosomal recessive disorder. The CMMRD syndrome is associated with a poor prognosis, and it is guided by biallelic mutations in DNA mismatch repair genes and has variable presentations. The patients of this syndrome are expected to have more than one malignancy starting commonly with hematological malignancies and brain tumors in the first decade of life, followed by colorectal cancers in the second decade, and then other tumors in early adulthood [1-4].

Based on the North American/Australian Registry, the CMMRD incidence in children of unrelated parents is about 1:10,00,000, to be substantially higher in children of consanguineous parents. A limited number of cases are reported in the literature concerning CMMRD syndrome (around 200 cases) with a limited number of rectal cancer cases [4,5]. To the best of our knowledge, only two cases with CMMRD syndrome were reported from the Gulf region, but our case is the first rectal cancer associated with CMMRD syndrome [6,7]. Therefore, we report a rare, very interesting, and unusual case of advanced low rectal cancer in a young female which was found to be related to CMMRD.

## Case presentation

**Figure 1:**
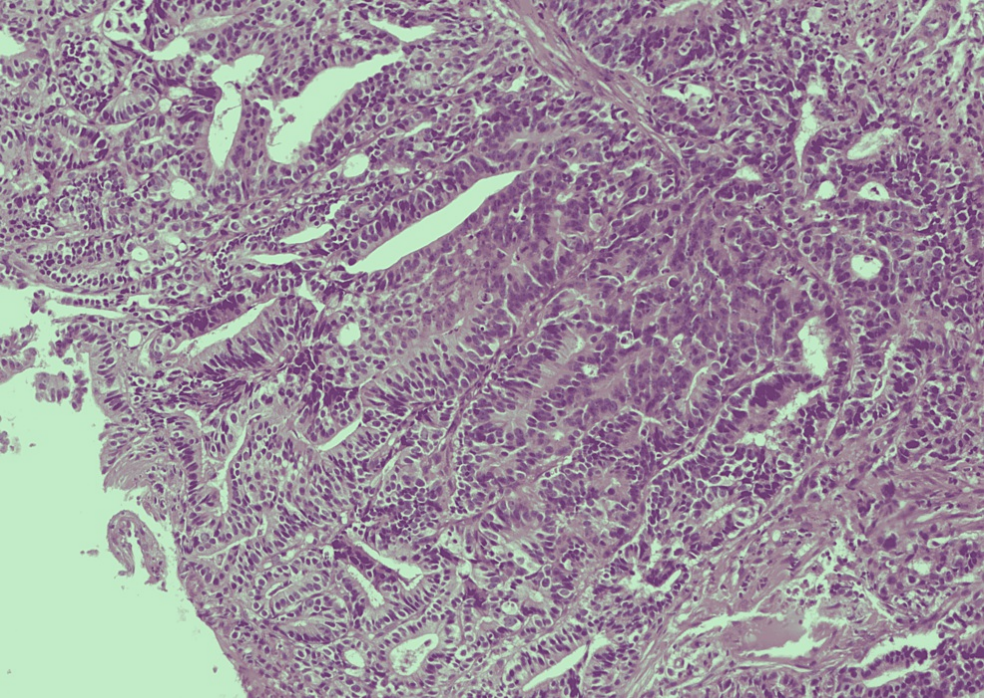
Histopathology of rectal biopsy. Light microscopy photographs of the tumor biopsy show glands forming carcinoma (hematoxylin and eosin stain; x100).

A 13-year-old Saudi female, born to non-consanguineous healthy parents and initially having an unremarkable medical and surgical history, referred to our colorectal surgery clinic as a case of advanced rectal cancer with a significant family history of malignancy (grandfather died at the age of 70 with colorectal cancer, and the first sibling died from brain tumors) for proper assessment and management. Initially, about eight months prior to presentation, she experienced a decreased oral intake and persistent diarrhea for almost one week followed by noticing a retractable bulging mass coming from her anus which made her afraid and hide the issue from her parents. About four to five months later, the patient started to have bloody diarrhea mixed with mucus associated with fatigue, loss of weight (14 kg in five months), and a progressive increase in the size of the mass. The family discovered the issue and sought medical advice where she was diagnosed as a case of hemorrhoids by a primary health care physician in a peripheral area. About two to three months later, the patient did not show signs of improvement which made the family seek another medical consultation, where she was examined and found to be pale, cachectic, and having café au lait spots all over her body and an ugly on/off bleeding mass protruding through the anus. The patient was investigated for the protruding mass and found to be anemic (Hgb 8.4 g/dL) for which she underwent a full colonoscopy which showed a big ulcerating mass that was located about 5 cm from the anal verge that was biopsied and reported as moderately to poorly differentiated invasive adenocarcinoma (Figure 1).

**Figure 2:**
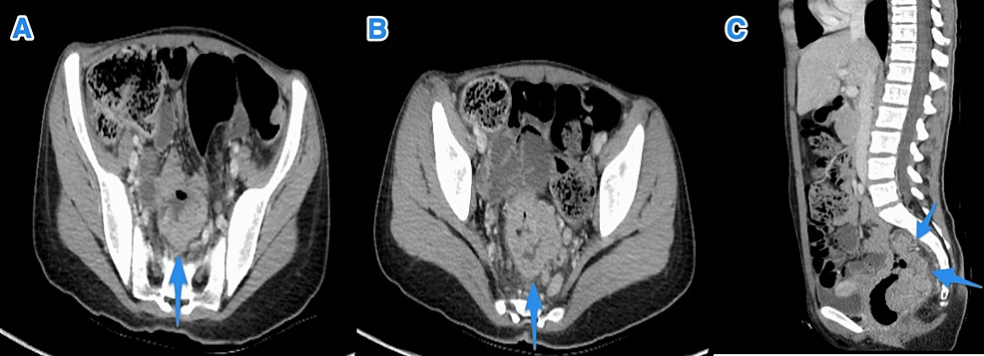
Baseline CT scan of the pelvis. CT scan of the pelvis axial (A and B) and sagittal (C) shows a mid rectal non-uniform wall thickening with a fungating mass (small arrow) associated with regional lymph nodes. Distally there is a large polypoidal enhancing intraluminal mass (large arrow) with a small pedicle posteriorly. There are no distant metastases.

When referred to our clinic, the case was reviewed, and examination revealed café au lait macules distributed all over her body parts of different sizes. Then full staging was done. Therefore, the carcinoembryonic antigen (CEA) level was 47 ng/mL, and computed tomography (CT) showed a 6.8 cm circumferential mid rectal mass that was extending all the way to the mesorectal fat. It also showed three colorectal polyps: two were located at the ascending colon and one was at the lower rectum with no evidence of an abdominopelvic metastasis (Figures 2A-2C). 

**Figure 3:**
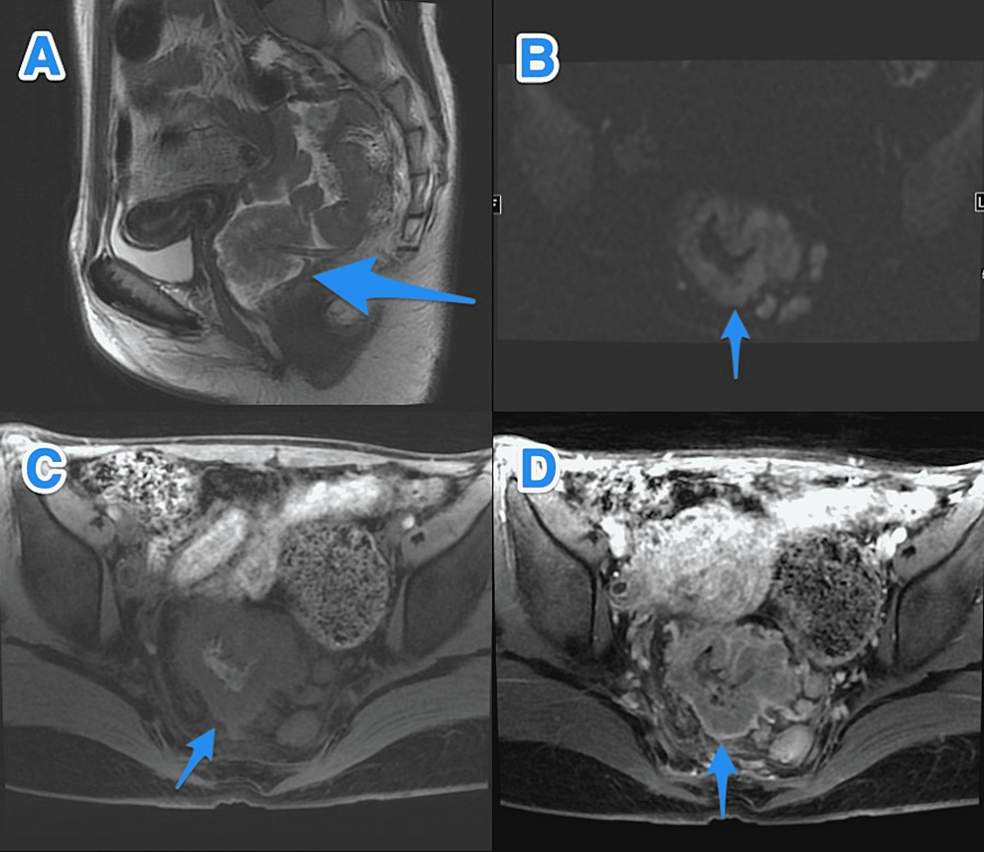
Baseline MRI study of the pelvis. MRI study of the pelvis sagittal T2 (A), axial DW (B), axial T1 (C), and axial T1 post-contrast (D) photographs show mid rectal circumferential wall thickening (small arrow) measuring 6.5 cm in length and 3.5 cm from the anorectal junction. This mass is invading the mesorectal fat at the left postero-lateral aspect with multiple large regional lymph nodes. Another large pedunculated polyp is seen in the lower rectum (large arrow), 2.2 cm from the anorectal junction with stalk arising from the posterior rectal wall. DW: diffusion-weighted

**Figure 4:**
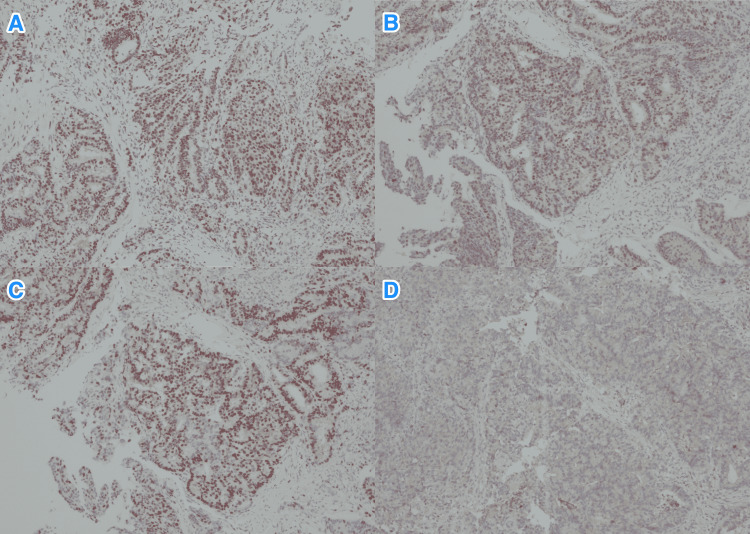
Immunohistochemistry staining of the rectal biopsy. Light microscopy photographs of immunohistochemistry staining study of the tumor biopsy cells show (A) nuclear positivity to MLH1 (x100), (B) nuclear positivity to MSH2 (x100), (C) nuclear positivity to MSH6 (x100), and (D) nuclear negativity to PMS2 (x100).

CT chest was negative for metastasis. Magnetic resonant imaging was done and showed a mid-to-lower rectal mass with multiple enlarged regional lymph nodes with a radiological staging of T3N2M0 (Figures 3A-3D). Further immunohistochemistry studies on the specimens showed intact MLH1, MSH2, and MSH6. However, PMS2 additionally showed a loss of nuclear expression increasing the probability of Lynch syndrome (Figures 4A-4D).

**Figure 5:**
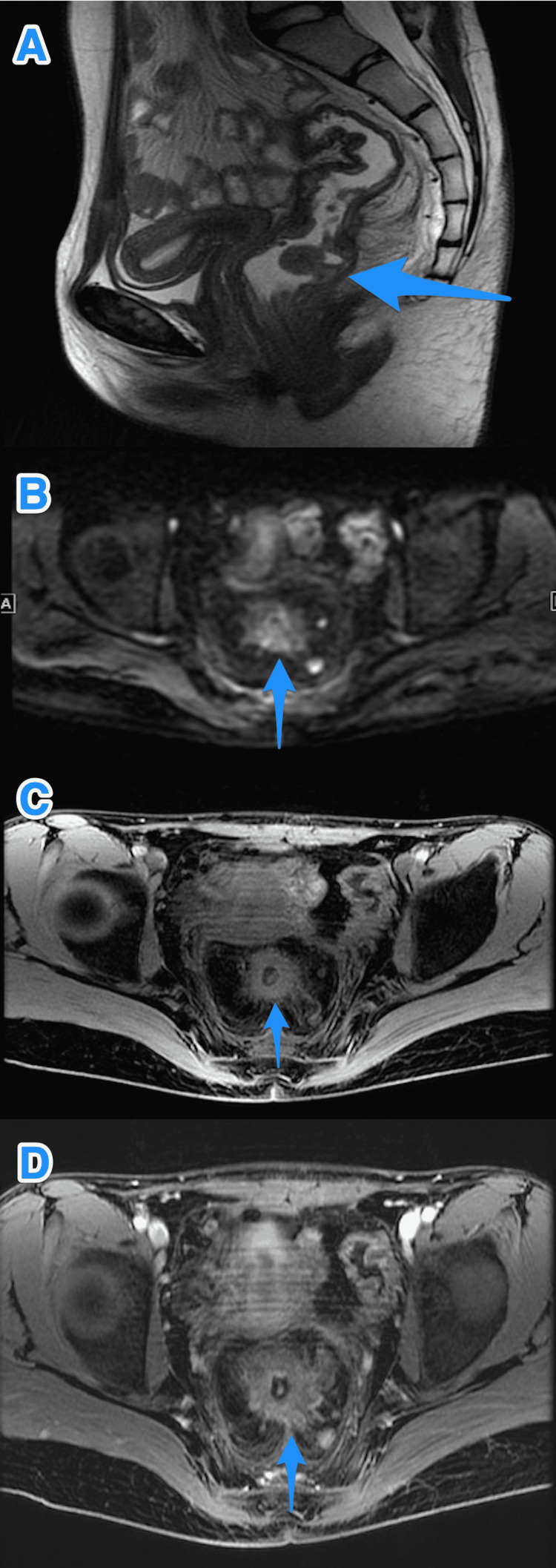
Follow-up (post-chemoradiotherapy) MRI study of the pelvis. MRI study of the pelvis sagittal T2 (A), axial DW (B), axial T1 (C), and axial T1 post-contrast (D) photographs shows significant interval improvement of the mid rectal tumor (small arrow) and mesorectal lymph nodes. The lower rectal polyp is also showing post-treatment response and size regression (large arrow). DW: diffusion-weighted

A positive K-RAS gene mutation was also found. Therefore, the case was discussed in the multidisciplinary tumor board and planned for genetic assessment, neoadjuvant chemotherapy (received three cycles of XELOX), then ovarian transposition and concurrent neoadjuvant chemoradiation therapy (received 5060 cGy in 23 fractions), and restaging prior to proceeding with surgery. The restaging MRI images showed a good response with about a 50% reduction in tumor size with no extension to mesorectal fat and interval regression of regional lymph nodes with a CEA level of 27 ng/mL (Figures 5A-5D).

**Figure 6:**
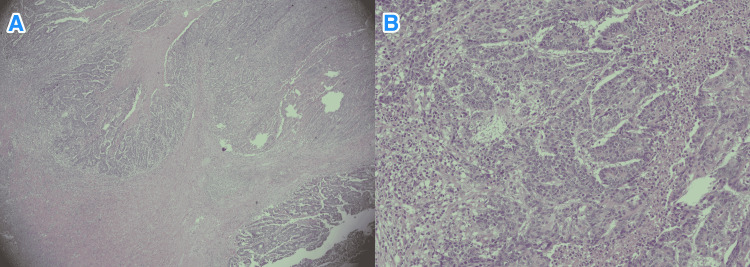
Histopathology of resected rectum. Light microscopy photographs of the tumor resection show glands forming carcinoma with focal necrosis (hematoxylin and eosin stain; {A} x20 and {B} x100).

Prior to surgery, a follow-up full colonoscopy was done, and it showed multiple polyps all over the colon except the cecum in addition to the already known rectal tumor. The patient underwent laparoscopic total proctocolectomy with ileal J-pouch creation and ileoanal anastomosis with temporary diverting loop ileostomy and an uneventful post-operative course. The histopathological examination of the resected specimen showed the same diagnosis with a pathological staging of T2N0M0 (Figures 6A, 6B).

The case was discussed post-operatively in the multidisciplinary tumor board and planned for counseling the family for adjuvant chemotherapy but the family elected not to proceed with the adjuvant therapy and just continue with follow-up. About three months later, the patient underwent an uneventful reversal of loop ileostomy, and she is currently on regular follow-up (six and 12 months already done) and surveillance with no recurrence or metastasis. The genetic assessment confirmed that the patient had CMMRD syndrome as her test analysis revealed a homozygous pathogenic variant in PMS2 (c.1376C>G). Therefore, the patient was placed on the regular screening protocol (Toronto protocol). After months of trials (her parents were in the denial phase), her siblings were scheduled for a cascade of testing and the parents for genetic testing and regular Lynch syndrome screening protocol (mostly, non-consanguineous parents with a heterogeneous mutation in PMS2).

## Discussion

Advanced malignancy and unexpected presentation in children or young generation are major challenges as in the current case. The literature still lacks information about some clinical disorders and syndromes like CMMRD, caused by a biallelic mutation in any of the four mismatch repair genes MSH2, MSH6 (20-30%), PMS2 (the most reported mutation, in over 60%, like in our case), and MLH1 if compared to LS. Since it is possible for both parents to have LS and it might be the situation in our case, the risk of CMMRD in their offspring will be 25% if compared to 50% for LS and 25% for having no LS. We found only two cases related to the CMMRD syndrome reported from our Gulf region, for instance, Glioblastoma (Kingdom of Saudi Arabia) and colon cancer (Qatar) [4,6-10].

It is important to notice that this patient is having multiple interesting findings including a significant history of familial malignancies, being born to non-consanguineous parents, the patient’s young age, and whether or not she presented with a prolapsing advanced rectal mass (rare presentation, most common Lynch syndrome-associated tumors) in addition to attenuated familial adenomatosis polyposis features and skin manifestation. Café au lait macules (CALMs) were reported to be a clinical sign in such syndrome which can be also a feature of neurofibromatosis type 1. Also, the presence of colorectal polyps was reported in the CMMRD syndrome commonly with the presence of 10 adenomas [2,11]. In 2021, the first established CMMRD diagnostic criteria were published which combined molecular diagnosis, ancillary testing, and clinical manifestation. In the past, we used to deploy the scoring system suggested in 2014 by the European Consortium Care for CMMRD (C4CMMRD) syndrome as a tool of investigation to determine which individual would undergo the genetic testing [10].

It is important to highlight that there is a major emotional and psychological impact of breaking such news about the presence of advanced cancer in a child with the possibility of having multiple cancers in the near future in addition to predisposing and testing other family members. We think it is important to use the genetic testing and screening tools for CMMRD with the other siblings as it has substantial help in better understanding of such syndrome as well as the early detection of tumors, therefore early intervention with the possibility of better outcomes. Up to date, the screening programs for CMMRD syndrome still lack efficacy as it's too rare and the literature lacks sufficient data to have guidelines to follow, and further studies are needed to determine the use of convenient tests at optimal intervals [12,13].

While genetics plays a major role in the new era of the medical field, in the diagnosis, surveillance, or even the management of the disorders or their consequences, the management of the CMMRD syndrome depends on the age of the patient as well as the type and stage of tumors. Therefore, we managed the current patient based on the guidelines for locally advanced rectal cancer as well as a consensus statement and recommendations by the US Multi-Society Task Force on Colorectal Cancer. The overall prognosis is poor for patients with CMMRD syndrome. Adjuvant chemotherapy in the presence of locally advanced rectal cancer might not change the prognosis as there has been inconsistency in its benefits. Education and increasing the awareness of parents and health workers in primary care or pediatricians play a major role in dealing with this syndrome [1,12,14-16].

## Conclusions

The number of syndromic diseases is rising; therefore, a detailed history, careful examination, proper utilization of genetics programs, and a high index of suspicion are the best keys in the diagnosis of serious health problems like CMMRD-related tumors during childhood. This is going to help in the proper screening, diagnosis, and management not only of the patients but also of the whole family.
